# Sequence Analysis of Toxin Gene–Bearing *Corynebacterium diphtheriae* Strains, Australia

**DOI:** 10.3201/eid2301.160584

**Published:** 2017-01

**Authors:** Christine J. Doyle, Adam Mazins, Rikki M.A. Graham, Ning-Xia Fang, Helen V. Smith, Amy V. Jennison

**Affiliations:** Queensland Health Forensic and Scientific Services, Brisbane, Queensland, Australia (C.J. Doyle, R.M.A. Graham, N.-X. Fang, H.V. Smith, A.V. Jennison);; Griffith University, Brisbane (A. Mazins)

**Keywords:** Diphtheria, Corynebacterium diphtheriae, Corynebacterium infections, diphtheria toxin, wound infection, bacterial infections, leg ulcer, skin diseases, bacteria, gram-positive bacterial infections

## Abstract

By conducting a molecular characterization of *Corynebacterium diphtheriae* strains in Australia, we identified novel sequences, nonfunctional toxin genes, and 5 recent cases of toxigenic cutaneous diphtheria. These findings highlight the importance of extrapharyngeal infections for toxin gene–bearing (functional or not) and non–toxin gene–bearing *C. diphtheriae* strains. Continued surveillance is recommended.

Pharyngeal diphtheria caused by toxigenic *Corynebacterium diphtheriae* is well-controlled in Australia due to a vaccine administered as part of the national immunization program. Rare cases of cutaneous and pharyngeal diphtheria have been reported in the country; however, the disease remains endemic in other regions of the world, and the potential for cases among travelers and their contacts remains (*1**–**4*). Historical data suggest that cutaneous diphtheria could be more contagious than respiratory diphtheria because environmental contamination from the skin is more common (*5*).

Detection of diphtheria toxin genes in either *C. ulcerans* or *C. diphtheriae* is notifiable in Queensland, regardless of the site of infection (*6*). Extrapharyngeal disease, such as cutaneous infection or endocarditis, caused by either toxigenic or nontoxigenic strains can be clinically notable, however, and is not prevented by vaccination (*4*). Our reference laboratory (Queensland Health Forensic and Scientific Services, Brisbane) receives isolates of *C. diphtheriae* and *C. ulcerans* from clinical laboratories in Queensland and surrounding areas for toxin gene testing. PCR is used to test for the presence of the toxin gene, which encodes for both subunits of the AB exotoxin. However, the functionality of the gene is not routinely examined (*7**,**8*). Previous studies in the United Kingdom and Russia have reported nonsense mutations in the toxin gene; those strains are described as nontoxigenic toxin gene–bearing (NTTB) (*9**,**10*). We aimed to identify potential mutations in the toxin gene in a selection of isolates in Australia, as well as describe the recent epidemiology of *C. diphtheriae* isolations in the local area after the annual number of isolates referred to the laboratory had increased 10-fold from 2012 (n = 9) through 2015 (n = 108).

During the 2-year period from July 1, 2013, until June 30, 2015, a total of 136 isolates of *C. diphtheriae* were referred to our laboratory for toxin gene screening; these isolates included 2 that were second isolations from patients, 2 and 3 months after the initial specimens were collected. Primary identification by diagnostic referring laboratories was confirmed by the presence of *dtxR* (*11*). We did not determine patient vaccination status, biotype of isolates, presence of co-infecting organisms, and antimicrobial susceptibility and treatment as part of this study.

Of the 136 isolates we received, 129 (95%) were from cutaneous wound swab specimens; 93 (72%) of 129 wounds were located on the lower limbs. Six isolates were respiratory system–associated, including 1 from the ear swab specimen of a patient with otitis media. Four isolates, including 1 nontoxigenic isolate from a blood culture, were from hospitalized patients, with the remainder presumed not to be. How this systemic case developed clinically is unknown. In most cases (71%), travel history or evidence on how the infection was acquired was not provided; however, when such information was given, tropical travel locations and injuries involving seawater or coral were typically noted. Isolates were collected from patients in both urban and rural areas.

Five of the 136 isolates had both A and B subunits of diphtheria toxin (*tox*), detected by multiplex PCR, all of which appeared to be functional by sequence analysis (*7*)*.* These 5 isolates were obtained from lower limb wound specimens from patients with a history of travel in a tropical travel area. Whole-genome sequencing with the Ion Torrent platform (Life Technologies, Grand Island, NY, USA) was performed on the 5 isolates with toxin genes detected by PCR and 1 historical isolate. We de novo assembled reads in Geneious R7 (Biomatters, Auckland, New Zealand) and used Ridom Seqsphere+ (Ridom GmbH, G Würzburg, Germany) to extrapolate in silico multilocus sequence typing (MLST) results and the sequences of diphtheria toxin repressor (*dtxR*) gene and *tox* genes. We also sequenced and analyzed the *tox* genes of an additional 8 historical *C. diphtheria*e isolates from our culture collection using methods described (*9**,**12*). MLST results for 3 historical isolates known to be related to each other were determined as described (*13*).

We analyzed a total of 14 *tox-*positive isolates (Table). All of those with MLST results had unique sequence types, except for the 3 known linked historical isolates. Novel sequences for *dtxR* and *tox* were submitted to GenBank (accession nos. KU869770–5). The novel *dtxR* sequences contained silent mutations and the novel frame-shift, missense, and/or nonsense mutations of the novel *tox* sequences. We predicted that 2 historical isolates would have nonfunctional *tox* genes, with single nucleotide deletions at positions 55 and 226 in fragment A, causing frame-shift mutations and premature stop codons at aa 38 and aa 92, respectively. These strains were isolated in 2006 or earlier and are considered to be NTTB strains. One of these strains has previously been reported as toxigenic; however, *tox* functionality was not assessed in that study by either sequence analysis or Elek testing (*14*).

**Table:** Analysis of tox gene–positive *Corynebacterium *diphtheria isolates, Australia*

Strain no.	GenBank accession no.		Predicted diphtheria toxin peptide sequence	MLST	Year isolated	Site	Patient travel history	Clinical note
	*dtxR *gene	*tox *gene						
WM960461361	Detected, NP	KX702990	Complete	NP	Unknown (1996 or prior)	Unknown	Unknown	Historical isolate
WM960431373	Detected, NP	KX702991	Complete	NP	Unknown (1996 or prior)	Unknown	Unknown	Historical isolate
WM00M102	Detected, NP	KX702992	Complete	NP	Unknown (2000 or prior)	Unknown	Unknown	Historical isolate
WM00M103	Detected, NP	KX702993	Truncated	NP	Unknown (2000 or prior)	Unknown	Unknown	Historical isolate
2006M0083	Detected, NP	KX702994	Complete	NP	2005	Lower limb	Indonesia	Coral cut
2006M2336	KU869770	KU869773	Truncated	ST379	2006	Lower limb	Indonesia	Coral cut
2011M2688	Detected, NP	KX702995	Complete	ST125	2011	Throat	No travel	Contact of carrier
2011M2777	Detected, NP	KX702996	Complete	ST125	2011	Throat	Papua New Guinea	Carrier
2011M2861	Detected, NP	KX702997	Complete	ST125	2011	Throat	No travel	Contact of case
2013M7922	KX702987	KU869774	Complete	ST381	2013	Lower limb	Papua New Guinea	Wound
2014M5840	KU869772	KX702998	Complete	ST243	2014	Lower limb	Cambodia	Wound
2014M7492	KX702988	KX702999	Complete	ST382	2014	Lower limb	Indonesia	Surf injury
2014M8143	KX702989	KU869775	Complete	ST120	2014	Lower limb	Unknown	Coral cut
2015M2871	KU869771	KX703000	Complete	ST380	2015	Lower limb	Solomon Islands	Wound

*MLST, multilocus sequence typing; NP, sequencing not performed.

The introduction of matrix-assisted laser desorption/ionization time-of-flight mass spectrometry as a routine identification tool in clinical microbiology laboratories has likely been a factor responsible for the continued increase in referral of isolates to our laboratory in recent years, possibly in addition to increased awareness after the fatal case of respiratory diphtheria in Australia in 2011. The 5 recent cases of functional toxin gene–bearing cutaneous *C. diphtheriae* infection more likely reflect an increase in testing cutaneous isolates rather than a true increase in incidence. Extrapharyngeal infections, particularly cutaneous, with both toxigenic and nontoxigenic strains are more common in this geographic region than is classical pharyngeal diphtheria, and their incidence is likely to have been historically underestimated. Repeat isolates from the same patient months after previous isolation reflect the chronic nature of cutaneous infection. This observation is also supported by most patients receiving care through outpatient settings. Any difference in the severity of disease caused by strains included this study is unknown, although we presume that functional toxin gene–bearing strains cause more severe disease.

Because of the theoretical possibility that NTTB stains and non–toxin gene–bearing strains could gain functional toxin expression by spontaneous mutation reversion or homologous recombination between different corynebacteriophages, these strains should be considered *tox* gene reservoirs (*9*). These strains also can cause systemic infections, as the blood culture isolate included in this study demonstrates. The genetic variation among the 5 recent functional toxin gene–bearing isolates indicates the absence of a particular circulating clone in the area. We recommend continued surveillance of *C. diphtheriae* and identification of NTTB strains.

## References

[1] Australian Government Department of Health. National Notifiable Diseases Surveillance System. [cited 2016 Jul 4]. http://www9.health.gov.au/cda/source/cda-index.cfm

[2] Abdul Rahim NR, Koehler AP, Shaw DD, Graham CR. Toxigenic cutaneous diphtheria in a returned traveller. Commun Dis Intell Q Rep. 2014;38:E298–300. 25631591 10.33321/cdi.2014.38.49

[3] Lingard S, Kleinschmidt S, Muttaiyah S, Appleton S, Playford G, Lampe G, et al. Fatal diphtheria: a case study. In: Proceedings of the Australian Society for Microbiology annual meeting, 2012. Brisbane (QLD, Australia): Australian Society for Microbiology; 2012. Abstract 724.

[4] Wilson APR. The return of *Corynebacterium diphtheriae*: the rise of non-toxigenic strains. J Hosp Infect. 1995;30(Suppl):306–12. 10.1016/0195-6701(95)90033-0 7560966

[5] Koopman JS, Campbell J. The role of cutaneous diphtheria infections in a diphtheria epidemic. J Infect Dis. 1975;131:239–44. 10.1093/infdis/131.3.239 805182

[6] Queensland Government. Public Health Regulation 2005. Office of the Queensland Parliamentary Counsel [cited 2016 Jul 4]. https://www.legislation.qld.gov.au/legisltn/current/p/pubhealr05.pdf

[7] Nakao H, Popovic T. Development of a direct PCR assay for detection of the diphtheria toxin gene. J Clin Microbiol. 1997;35:1651–5. 9196167 10.1128/jcm.35.7.1651-1655.1997 PMC229815

[8] Chenal A, Nizard P, Gillet D. Structure and function of diphtheria toxin: from pathology to engineering. J Toxicol. 2002;21:321–59. 10.1081/TXR-120014408

[9] Zakikhany K, Neal S, Efstratiou A. Emergence and molecular characterisation of non-toxigenic tox gene-bearing *Corynebacterium diphtheriae* biovar mitis in the United Kingdom, 2003-2012. Euro Surveill. 2014;19:20819. 10.2807/1560-7917.ES2014.19.22.20819 24925458

[10] Mel’nikov VG, Kombarova SI, Borisova OI, Volozhantsev NV, Verevkin VV, Volkovoĭ KI, et al. *Corynebacterium diphtheriae* nontoxigenic strain carrying the gene of diphtheria toxin [in Russian]. Zh Mikrobiol Epidemiol Immunobiol. 2004;1:3–7. 15024972

[11] Pimenta FP, Matias GAM, Pereira GA, Camello TCF, Alves GB, Rosa ACP, et al. A PCR for dtxR gene: application to diagnosis of non-toxigenic and toxigenic *Corynebacterium diphtheriae.* Mol Cell Probes. 2008;22:189–92. 10.1016/j.mcp.2008.01.001 18378421

[12] Mancini F, Monaco M, Pataracchia M, von Hunolstein C, Pantosti A, Ciervo A. Identification and molecular discrimination of toxigenic and nontoxigenic diphtheria *Corynebacterium* strains by combined real-time polymerase chain reaction assays. Diagn Microbiol Infect Dis. 2012;73:111–20. 10.1016/j.diagmicrobio.2012.02.022 22494559

[13] Bolt F, Cassiday P, Tondella ML, Dezoysa A, Efstratiou A, Sing A, et al. Multilocus sequence typing identifies evidence for recombination and two distinct lineages of *Corynebacterium diphtheriae.* J Clin Microbiol. 2010;48:4177–85. 10.1128/JCM.00274-10 20844217 PMC3020856

[14] May ML, McDougall RJ, Robson JM. *Corynebacterium diphtheriae* and the returned tropical traveler. J Travel Med. 2014;21:39–44. 10.1111/jtm.12074 24383653

